# Breast cancer stromal elastosis is associated with mammography screening detection, low Ki67 expression and favourable prognosis in a population-based study

**DOI:** 10.1186/s13000-014-0230-8

**Published:** 2014-12-19

**Authors:** Ying Chen, Tor A Klingen, Elisabeth Wik, Hans Aas, Einar Vigeland, Knut Liestøl, Øystein Garred, Jan Mæhlen, Lars A Akslen, Jon Lømo

**Affiliations:** Department of Pathology, Vestfold Hospital, Tønsberg, Norway; Centre for Cancer Biomarkers CCBIO, Department of Clinical Medicine, Section for Pathology, University of Bergen, Bergen, Norway; Department of Pathology, Haukeland University Hospital, Bergen, Norway; Department of Surgery, Vestfold Hospital, Tønsberg, Norway; Department of Radiology, Vestfold Hospital, Tønsberg, Norway; Institute of Informatics, University of Oslo, Oslo, Norway; Department of Pathology, Oslo University Hospital, 0424 Oslo, Norway; Department of Pathology, Akershus University Hospital, Lørenskog, Norway

**Keywords:** Breast cancer, Mammography screening, Elastosis, Ki67

## Abstract

**Background:**

Mammography screen-detected breast cancers have a better prognosis than predicted from established prognostic markers. A search for additional features that are characteristic for these tumours and their prognosis is needed to reduce overtreatment, a recognized challenge in breast cancer patient management today. Here, we have investigated the occurrence and importance of tumour elastosis.

**Methods:**

We performed a population based retrospective study of breast cancers detected in the Norwegian Breast Cancer Screening Programme in Vestfold County during 2004–2009. In total, 197 invasive screen-detected cancers and 75 interval cancers in patients aged 50–69 years were compared with regard to standard clinico-pathological parameters and tumour shape, as well as ER, PR, HER2 and Ki67 expression. In particular, the presence of elastotic material in tumours was graded on a 4-tiered scale (score 0–3).

**Results:**

Screen-detected cancers had a significantly higher content of stromal elastosis than interval cancers (p < 0.001). High content of elastosis (score 3) correlated strongly with stellate tumour shape, low histological grade, and ER+/HER2- status. Further, high elastosis score was significantly associated with lower Ki67 expression. In survival analyses, cases with high elastosis demonstrated increased recurrence free (p = 0.03) and disease-specific survival (p = 0.11) compared to cases with low elastosis.

**Conclusion:**

There is a strong correlation between the presence of tumour elastosis, stellate tumour shape and mammography detection of breast cancers. To our knowledge, this is the first time elastosis has been studied in relation to breast cancer detection method. Presence of elastosis is associated with low tumour cell proliferation (Ki67) and a good prognosis.

**Virtual Slides:**

The virtual slide(s) for this article can be found here: http://www.diagnosticpathology.diagnomx.eu/vs/13000_2014_230

## Background

The value of population-wide mammography screening is controversial [1,2]. Thus, the introduction of mammography screening for breast cancer has been associated with up to 50% incidence increase in invited age groups [3-5]. Both tumour size and other prognostic factors are more favourable for screened cancers than for symptomatic (non-screened) tumours [6]. This is expected since the aim of screening is to detect cancers early when cure is possible. However, follow-up studies from different countries indicate that screen-detected cancers have more low-grade features and better outcome than predicted from tumour size, histological grade, and lymph node status included in the Nottingham Prognostic Index [7,8]. Furthermore, molecular subtyping of tumours can only partly account for the difference [9-12]. Therefore, a search for additional histological or molecular characteristics of screen-detected cancers with an especially favourable prognosis is needed. Their identification could result in less overtreatment, which is a known challenge in breast cancer patient management today [13].

Breast cancers are morphologically diverse and show a wide spectrum of growth patterns and features such as expression of ER, PR, HER2 and tumour cell proliferation. Recently, studies have also focused on the stroma (microenvironment) surrounding tumour cells and providing growth conditions and support for malignant cells. The tumour stroma consists of several components, including extracellular matrix (composed of various proteoglycans and fibrous proteins) [14], certain mesenchymal cells and leukocytes, as well as the vasculature. Both histochemical and molecular studies of breast cancer stroma have been conducted [15-17], and one striking finding in many tumours is the large aggregates of elastin fibres, known as elastosis. This feature is also observed in radial scars, a benign condition mimicking breast cancer on mammograms [18].

Elastin is normally expressed in significant quantities in skin, lung, cartilage, and large arteries, and it is produced by fibroblasts, smooth muscle cells and chondrocytes as a 72 kDa precursor tropoelastin, which is secreted into the extracellular microenvironment. This protein is cross-linked by lysyl oxidase (LOX) and complexes with several smaller proteins, including fibrillin, to form large structures with unique elastic properties [19]. In breast cancer, elastin is observed as both individual fibres in the stroma and large aggregates around ducts or small blood vessels. The origin of the elastotic material has been shown to be stromal cells like fibroblasts and myofibroblasts [20], but also the carcinoma cells [15,21]. Further, a 67 kD elastin receptor (EBP/S-gal) has been reported [22,23], and receptor stimulation can affect cell proliferation, adhesion, and chemotaxis. In addition, elastin can be cleaved into small peptide fragments, which can affect different cellular processes including apoptosis, chemotaxis, and metastasis [23,24].

Elastosis was first studied in breast cancer decades ago by Shivas & Douglas and others, showing that tumours with large amounts of elastotic material had a better prognosis [25,26]. Following this study, several groups confirmed the correlation between elastosis and improved breast cancer survival [27-29]. Further, there is a correlation between elastosis and estrogen receptor expression as well as response to anti-hormonal therapy [30,31]. On this background, the aim of our study was to establish whether there is a significant difference in elastosis content between screen detected and interval breast cancers, and whether elastosis is a marker of cancers detected by mammography and a possible mediator of their good prognosis. The study was based on material from the population-based Norwegian Breast Cancer Screening Programme.

## Methods

### Study population

Patients were included from Vestfold County in Eastern Norway. Vestfold comprises 5% of the Norwegian population with around 230,000 inhabitants. The Norwegian Breast Cancer Screening Programme involves biannual mammography in the age-group 50–69 years, and was implemented in this county in 2004. The mean age of the actual patients at the time of diagnosis was 60 years (range 49–70 years). A total of 37,977 women participated during the study period March 2004 – June 2009 with attendance rates of 71% and 76%, respectively, during the first two screening rounds. During this period, a total of 285 patients with 202 invasive screen-detected cancers and 83 invasive interval tumours were diagnosed during the prevalent and subsequent rounds. The term interval cancer refers to a breast cancer diagnosed between two screening sessions. Ten patients (3 screening and 7 interval) were excluded because only core biopsies were available from these locally advanced tumours; the evaluation of elastosis content was limited to cases where standard sections were available (cases treated by primary surgery). In addition, 3 other patients were excluded: 1 screen-detected cancer had no residual tumour tissue for further investigation; 1 screen-detected case was diagnosed as a malignant phyllodes tumour, and 1 patient with interval cancer suffered from multiple metastases at the time of diagnosis, and no biopsy or surgery of the breast was performed. Four patients (two screening-patients and two interval-patients) had simultaneous tumours in both breasts. To represent the case for this particular study, the Nottingham Prognostic Index (NPI) was used for three of them, to select the tumour with charateristics of the worst prognosis. The fourth patient had an identical NPI in both tumours; the tumour with highest Ki67 score was subsequently selected for our study. Thus, a total of 272 patients with 197 from the screening group and 75 from the interval group were included for this population-based study. Clinical data (stage and survival) were recorded from patient journals (last clinical status June 2013). The study was approved by the Regional Ethics Committee of Eastern Norway (registration number S-08685d).

### Histopathological data

All cases were re-examined by microscopy by one of the authors (T.A.K.) by using standard HES stained sections. Tumours were classified as either ductal, lobular or other types of carcinomas. Histological grading was done according to the Nottingham criteria [32]. Tumour diameter was measured microscopically in mm, and lymph node status was included from the pathology report. For this study, all tumours underwent standard immunohistochemical staining for ER, PR and HER2. For ER and PR, positivity was defined as staining in ≥ 10% of the tumour cell nuclei. In equivocal HER2 cases (2+ according to the HerCep test criteria), in situ hybridization by FISH analysis was done. Amplification was defined as a HER2/Chromosome 17 ratio of ≥ 2 [33].

#### Ki67

Tissue microarray slides from the tumour set were used for Ki67 evaluation. The slides were dewaxed with xylene/ethanol before microwave antigen retrieval for 20 min in TRS buffer (pH9). The slides were incubated for 30 min with a monoclonal Ki67 antibody (M7240, clone MIB-1) (Dako), diluted 1:100. The staining was performed using the EnVision-labelled polymer method. 500 tumour cell nuclei were counted, and the Ki67 positive fraction was calculated.

Further histological evaluation was done in conjunction by two authors (Y.C. and J.L.) in a blinded manner for the following parameters:

#### Tumour elastosis

The amount of elastotic stroma in the tumour, which was evaluated in cases with standard sections available, was graded by microscopy in a semiquantitive manner from 0 – 3, according to Shivas & Douglas [25]. This method refers to elastin-stained microscopic slides, but elastosis can also easily be recognized in standard HES slides as a deposit of grey, fibrillary material. In grade 0, elastosis was absent; in grade 1, small deposits (single elastin fibrils or a thin rim of elastosis around ducts) were present; in grade 2, thicker zones of elastosis were found; in grade 3, large deposits dominated substantial areas of the tumour. Both evaluation methods for elastin were used in the present study. Elastosis was graded without knowledge of other features or method of detection. In some of the statistical analyses, elastosis was divided into two categories, low (grade 0 to 2) and high (grade 3). Morphologically, this cut-off point corresponds to cases with no or limited elastosis (grade 0–2) compared to cases with extensive elastosis (grade 3).

#### Tumour shape

The contour shape of the tumour was evaluated in whole tumour slides. If tumour processes (containing tumour cells) were radiating into surrounding tissue, the tumour shape was classified as stellate [34,35]. Tumours that lacked these features were classified as round. These tumours typically had pushing border growth.

#### Necrosis

Presence of necrotic areas in the infiltrating tumour tissue (not necrosis in DCIS areas) was noted. A necrotic focus was defined as a confluent area of necrosis detectable at intermediate magnification (x40), containing at least 10–15 necrotic cells [36].

#### Fibrotic focus

The presence of a large collagenous area devoid of carcinoma cells, usually in the center of the tumour, was noted. This has previously been linked to worse prognosis in breast cancer [37,38]. A fibrotic focus does not contain elastosis.

### Statistical analysis

All statistical analyses were performed with IBM SPSS Statistics, version 21.0 (Armonk, NY: IBM Corp.). Two-sided p-values of <0.05 were considered statistically significant. Associations between different categorical variables were assessed by Pearsons χ^2^ test or Kendall´s test. Mann–Whitney U test was used for analysis of age, tumour diameter and Ki67 between categories. Univariate survival analyses of time to death due to breast cancer (disease specific survival) and time to recurrence for patients without metastases at the time of diagnosis (recurrence free survival), were performed using the Kaplan–Meier method (log-rank test). Entry date was the time of diagnosis. Patients who died from other causes were censored at the date of death in the analyses of disease specific survival. For statistical analyses, tumour elastosis was dichotomized; high elastosis (grade 3) and low elastosis (grade 0–2). A total of 272 patients were accessible for survival analysis in the current study.

## Results

### Elastosis content

**Figure 1:**
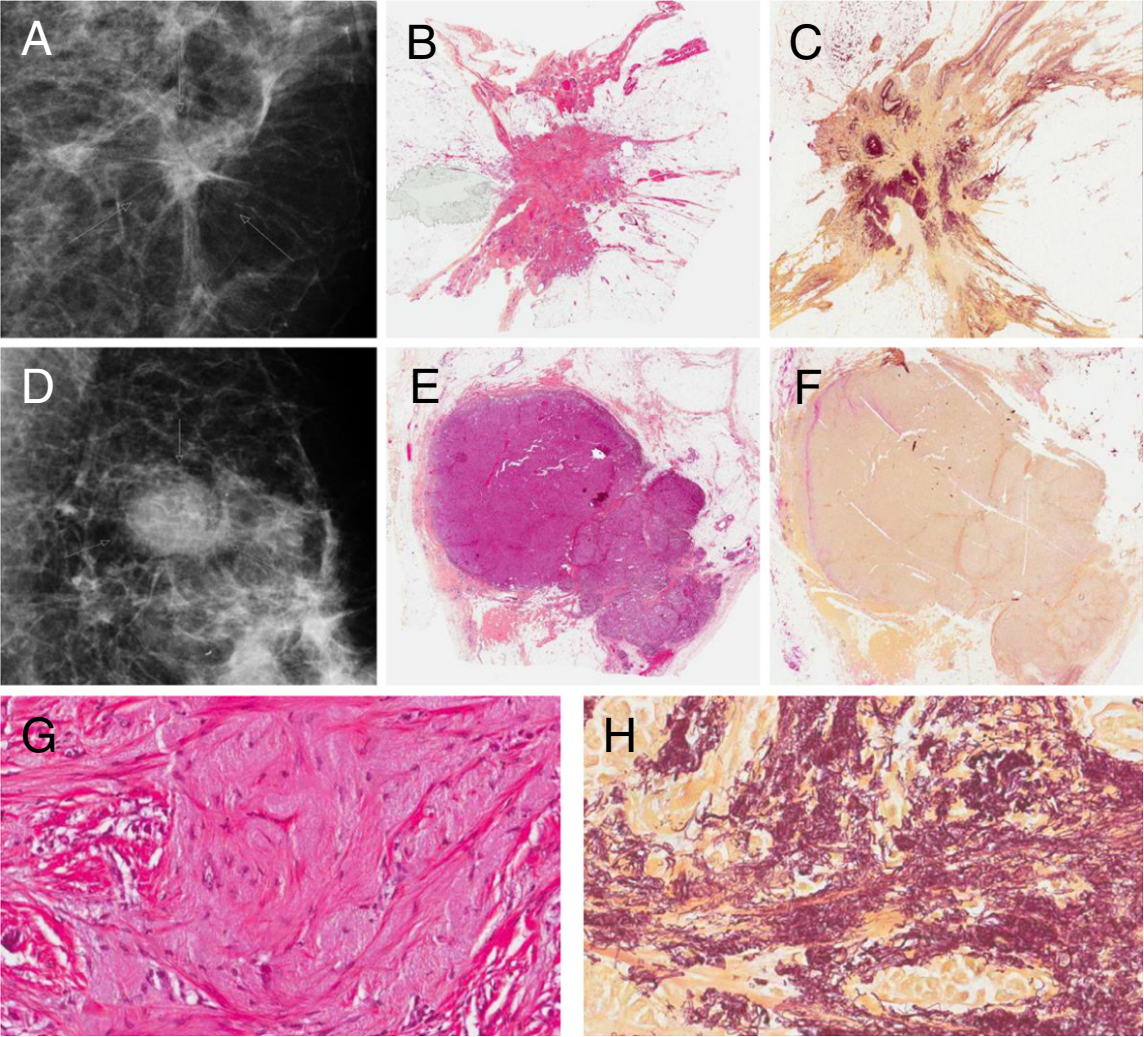
**Histological and radiological images of screen-detected and interval breast cancer.** Upper row: Radiological **(A)** and histological whole tumour images of a screen-detected cancer with stellate shape and abundant stromal elastosis (grade 3) (HES staining in **B**; elastin-staining in **C)**. Middle row: Comparable images of an interval cancer with rounded, pushing-border contour and no stromal elastosis (grade 0) **(D-F)**. Magnification x 5. Lower row: Higher magnification (x200) of histological slides from the screening cancer in the upper row. **G**, HES-staining. **H**, elastin-staining.

Figure 1 shows tumours with typical elastosis grade 3 and 0, respectively. Table 1 shows a comparison of elastin content by HES sections and elastin special stain in all samples. Although significantly correlated (p < 0,001), some discordant cases were observed. Elastin staining colours the fibres black which explains a higher sensitivity than visual recognition of the grey material in HES-slides. Thus, a proportion of HES negative slides were 1+ by elastin staining, and likewise for HES 1+ being 2+ with elastin stain. There was almost complete concordance between HES 3+ and elastin-stain 3+ cases.

**Table 1:** Methods of elastin fiber visualization: HES staining versus elastin special stain*

	**Elastosis grade using HES stain**				
**Elastin stain**	**0**	**1**	**2**	**3**	**Total**
0	56	1	0	0	57
1	35	54	2	0	91
2	1	23	51	0	75
3	0	0	4	45	49
Total	92	78	57	45	272

Elastosis was graded semiquantitatively from 0–3 using either stain.

*p-value < 0.001 for comparison (Kendall´s test).

### Characteristics of tumours detected by screening versus interval cancers

Table 2 presents the basic clinico-pathological features and Table 3 the special histopathological evaluation of breast carcinomas in relation to detection method in the study population. As expected, histological grade was significantly higher in interval cancers; the proportion of grade 3 tumours was 14% in the screen-detected group and 40% in the interval cancer group. Tumour size and frequency of lymph node involvement were moderately higher for the interval versus screen-detected cancers. ER positivity was more frequent in screen-detected cancers, whereas PR and HER2 expression were not significantly different in this study.

**Table 2:** **Breast cancer detection method in association with clinical and histopathological characteristics**

	**Detection method**		
**Marker**	**Screening**	**Interval**	
	n (%)*	n (%)*	p-value**
**Age**	60.0 (5.7)	60.0 (5.7)	NS
**Tumour diameter**	14.0 (9.9)	16.0 (13.1)	NS
**Histological type**			NS
Ductal	159 (81)	61 (81)	
Lobular	23 (12)	10 (14)	
Other	15 (7)	4 (5)	
**Histological grade**			<0.001
Grade 1	63 (32)	12 (16)	
Grade 2	107 (54)	32 (43)	
Grade 3	27 (14)	31 (41)	
**Nodal status**			NS
N0	136 (69)	45 (60)	
N1+	61 (31)	30 (40)	
**ER**			0.001
Positive	180 (91)	57 (76)	
Negative	17 (9)	18 (24)	
**PR**			NS
Positive	133 (67)	48 (64)	
Negative	64 (33)	27 (36)	
**HER2**			NS
Negative	184 (93)	65 (87)	
Positive	13 (7)	10 (13)	
**Ki67 (median)**	10 (12,9)	12,0 (17,5)	NS

*Number of cases (n and %) is given except for age, tumour diameter and Ki67, where median and standard deviation (SD) is given.

**Pearson's Chi-square test except for age, tumour diameter and Ki67 where Mann–Whitney U test was applied.

**Table 3:** **Breast cancer detection method in association with tumour shape and stromal characteristics**

	**Detection method**		
**Marker**	**Screening**	**Interval**	p-value**
	n (%)*	n (%)*	
**Tumour shape**			0.001
Star	143 (73)	37 (51)	
Round	54 (27)	36 (49)	
**Elastosis** ^**#**^			<0.001
0	53 (27)	39 (52)	
1	56 (29)	22 (29)	
2	46 (23)	11 (15)	
3	42 (21)	3 (4)	
**Fibrotic focus**			NS
No	178 (90)	64 (87)	
Yes	19 (10)	10 (13)	
**Necrosis**			0.012
No	183 (93)	62 (83)	
Yes	14 (7)	13 (17)	

*Number of cases (n and %).

**Pearson`s chi-square test.

^#^Graded on HES-slides.

Further, screen-detected cancers were significantly associated with star-shape (Figure 1) and absence of necrosis (Table 3). Also, high elastosis content was more frequent in the screen-detected cancers; 44% of these tumours were 2+ or 3+ compared with 19% among interval cancers; 52% of interval tumours lacked elastosis altogether, compared with only 27% of screen-detected cancers. Presence of a fibrotic focus was not significantly different in the two groups.

### High elastosis content associates with clinico-pathological characteristics of good prognosis

**Figure 2:**
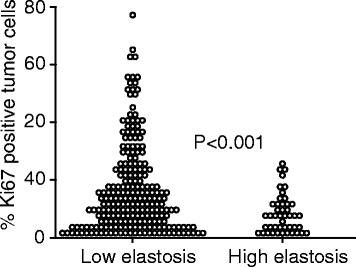
**High elastosis is significantly associated with low proportion of Ki67 positive tumour cells.** The relationship between high/low elastosis and % Ki67 positive tumour cells are demonstrated in the dot-blot.

High stromal elastosis was associated with ER and PR expression and HER2 negativity (Table 4). Also, tumours with high elastosis content were significantly associated with stellate tumour shape and absence of fibrosis and necrosis (Table 4). There was significantly more frequent low-grade tumours in the high-elastosis cases, compared to tumours with low elastosis. High elastosis score was also significantly associated with lower tumour cell proliferation by Ki67 expression (Figure 2 and Table 4). Tumour size, histological type, lymph node status, and distant metastasis did not show a significant correlation with elastosis.

**Table 4:** **Correlation between elastosis (high/low) and clinico-pathological features**

	**Elastosis (HES slides)**		
**Marker**	**Low elastosis (0–2)**	**High elastosis (3)**	
	n (%)*	n (%)*	p-value**
**Age**	60.0 (5.7)	63.0 (5.2)	NS
**Tumour diameter**	15,0 (11.3)	14,0 (8.7)	NS
**Histological type**			NS
Ductal	184 (81)	36 (80)	
Lobular	26 (12)	7 (16)	
Other	17 (7)	2 (4)	
**Histological grade**			<0.001
Grade 1	53 (23)	22 (49)	
Grade 2	117 (52)	22 (49)	
Grade 3	57 (25)	1 (2)	
**Nodal status**			NS
N0	147 (65)	34 (76)	
N1+	80 (35)	11 (24)	
**ER**			0.005
Positive	192 (85)	45 (100)	
Negative	35 (15)	0 (0)	
**PR**			0.04
Positive	145 (64)	36 (80)	
Negative	82 (36)	9 (20)	
**HER2**			0.03
Negative	204 (90)	45 (100)	
Positive	23 (10)	0 (0)	
**Ki67**	12,0 (15,2)	7,0 (6,6)	<0.001
**Tumour shape**			<0.001
Star	136 (60)	44 (98)	
Round	89 (40)	1 (2)	
**Fibrotic focus**			0.011
No	197 (87)	45 (100)	
Yes	29 (13)	0 (0)	
**Necrosis**			0.015
No	200 (88)	45 (100)	
Yes	27 (12)	0 (0)	

*Number of cases (n and %) is given except for age, tumour diameter and Ki67, where median and standard deviation (SD) is given.

**Pearson's chi-square test except for age, tumour diameter and Ki67 where Mann–Whitney U test was applied.

### Survival analysis

The median follow-up period was 71 months (range 2–117 months). Among 272 patients finally included, distant metastases or local tumour recurrence were observed at follow-up in 31 (12 %), and 22 patients (8 %) died of breast cancer.

**Figure 3:**
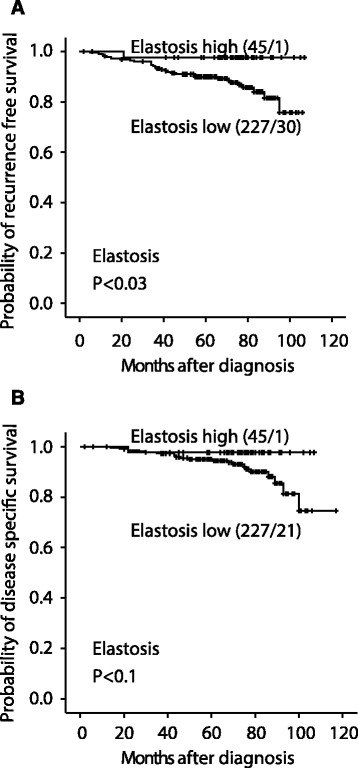
**Elastosis is associated with survival in breast cancer.** Low elastosis (evaluated by HES-section) associates with reduced recurrence-free **(A)** and disease specific **(B)** survival. For each category, the number of cases is given followed by the number of breast cancer recurrences or deaths.

In univariate survival analyses, high elastosis content was significantly associated with longer recurrence-free survival as compared to cases with low elastosis (5-year survival 98% and 90 %, respectively, Figure 3A). High elastosis was also associated with longer disease specific survival, although of borderline significance (98% and 96%, respectively, Figure 3B).

## Discussion

Breast cancers detected by mammography screening have a particularly good prognosis, better than predicted by standard clinico-pathological features. A tumour stroma rich in elastin fibre aggregates (elastosis) is a frequent finding in breast cancer. By comparing screen-detected and interval cancers in a population-based material from the Norwegian Breast Cancer Screening Programme, we here found a strong correlation between screening detection and tumour elastosis. High content of elastosis in turn showed strong covariation with other predictors of good prognosis such as low histological grade, hormone receptor expression, HER2 negative tumours, and low tumour cell proliferation by the Ki67 index. Also, significantly improved recurrence free survival was observed in tumours with high elastin content. To our knowledge, this is the first time elastosis content is studied in relation to breast cancer detection method.

Tumour elastosis was associated with stellate shape, which is frequent in lesions detected by mammography. Mammographic spiculation has previously been found to correlate with good prognostic factors [39,40]. Moreover, one study showed that mammographic spiculation was an independent prognostic factor for screen-detected breast cancer [39]. In another study, not stratified by detection method, small breast cancers (less than 15 mm) with stellate mammographic pattern had a better survival [41]. A previous study found that stellate ductal carcinomas (termed “scar cancer”) were related to markers of good prognosis such as small tumour size and hormone receptor positivity [34]. Since mammography detection of malignancy is partly based on the recognition of a stellate tumour pattern, this detection method appears to be biased towards low-grade and good-prognosis tumours. Because of its association with stellate morphology, tumour elastosis may be one factor that mediates this favourable prognosis.

In contrast to screen-detected breast cancer in our study, interval cancers typically had low or absent elastosis content. They tended to have a rounded shape, and displayed necrosis more frequently, the latter reflecting their more aggressive phenotype. These tumours also had higher histological grade and Ki67 index and were more often hormone receptor negative and HER2 positive, which is in accordance with previous studies [12].

What is the biological significance of aggregation of large quantities of elastin fibres in breast cancer stroma? Is there a mechanistic link between the presence of elastin and a better prognosis? The angiostatic molecule endostatin has been reported to accumulate on elastin fibres [42], and this could reduce angiogenesis and hence tumour growth and spread. In contrast, effects could be related to elastin itself, the elastin receptor, or elastin derived peptides. Some elastin-related effects may be mediated by elafin, an inhibitor of elastase, which recently has been shown to be a positive prognostic factor in breast cancer [43]. Other recent studies have addressed the mechanical properties of tumour tissue, establishing a correlation with aggressive behavior in collagen-rich tumours [44]. Hypothetically, a high content of elastin may affect the mechanical properties and prognosis in the opposite direction. The elucidation of these mechanisms needs further investigation.

Mammography screening has been criticized for only a small reduction in breast cancer mortality, although studies differ in their estimates [45-47]. At the same time, screening significantly increases the incidence of invasive breast cancer, in some studies by up to 50% in the invited age group. This has lead to the hypothesis that a subgroup of screen-detected tumours would never manifest clinically because they remain dormant or regress spontaneously [3]. It is tempting to speculate that large areas of elastosis could be relevant in this context. At least, elastosis is probably not a favourable microenvironment for tumour growth and spread.

## Conclusions

Our results indicate a strong correlation between the presence of tumour elastosis and mammography detection of breast cancers, and there was also an association with stellate tumour shape, a frequent detection criterion by mammography. The presence of elastosis was associated with low-grade tumours and good prognosis. Whether and how elastosis is mechanistically involved in tumour development and progress requires further study.

## Abbreviations

DSS: Disease specific survival
ER: Estrogen receptor
HES: Haematoxylin eosin safran stain
PR: Progesterone receptor
RFS: Recurrence free survival
